# Constructing Physics-Informed Neural Networks with Architecture Based on Analytical Modification of Numerical Methods by Solving the Problem of Modelling Processes in a Chemical Reactor

**DOI:** 10.3390/s23020663

**Published:** 2023-01-06

**Authors:** Dmitriy Tarkhov, Tatiana Lazovskaya, Galina Malykhina

**Affiliations:** 1 Department of Higher Mathematics, Peter the Great St. Petersburg Polytechnic University, 29 Polytechnicheskaya Str., 195251 Saint Petersburg, Russia; 2 Scientific and Technological Centre (STC) “Mathematical Modelling and Intelligent Control Systems”, High School of Cyber-Physical Systems and Control, Peter the Great St. Petersburg State Polytechnic University, 29 Polytechnicheskaya Str., 195251 Saint Petersburg, Russia

**Keywords:** PINN, numerical method, analytical modification, shooting method, data-driven modelling, chemical reactor, engineering applications, intelligent systems, multi-fidelity, surrogate modelling

## Abstract

A novel type of neural network with an architecture based on physics is proposed. The network structure builds on a body of analytical modifications of classical numerical methods. A feature of the constructed neural networks is defining parameters of the governing equations as trainable parameters. Constructing the network is carried out in three stages. In the first step, a neural network solution to an equation corresponding to a numerical scheme is constructed. It allows for forming an initial low-fidelity neural network solution to the original problem. At the second stage, the network with physics-based architecture (PBA) is further trained to solve the differential equation by minimising the loss function, as is typical in works devoted to physics-informed neural networks (PINNs). In the third stage, the physics-informed neural network with architecture based on physics (PBA-PINN) is trained on high-fidelity sensor data, parameters are identified, or another task of interest is solved. This approach makes it possible to solve insufficiently studied PINN problems: selecting neural network architecture and successfully initialising network weights corresponding to the problem being solved that ensure rapid convergence to the loss function minimum. It is advisable to use the devised PBA-PINNs in the problems of surrogate modelling and modelling real objects with multi-fidelity data. The effectiveness of the approach proposed is demonstrated using the problem of modelling processes in a chemical reactor. Experiments show that subsequent retraining of the initial low-fidelity PBA model based on a few high-accuracy data leads to the achievement of relatively high accuracy.

## 1. Introduction

A classical approach to modelling real objects consists of two steps. In the first step, based on the analysis of known facts about physical (and other) processes taking place in an object, a model is constructed in the form of a differential equation (system of equations) and additional conditions (initial, boundary, etc.). In the second step, the model obtained is investigated using numerical methods. The final model in the form of a table of numbers allows for drawing the necessary conclusions about the object’s behaviour, plotting graphs, predicting their dynamics, etc. If it turns out that the operation data differ significantly from the model constructed, there is a need to return to the first stage and build a more accurate differential model, then rebuild the table of numerical solutions. The differential equation solvers can be computationally costly compared to the case when it is possible to operate a general parametric analytical model. Moreover, if such an approach does not lead to success, it is necessary to build a model of the object directly from measurement data that is not the strength of classical numerical methods. The same problem arises in engineering when it is required a thorough analysis of the model performance under various parameters. The underlying equations have to be solved for a large amount of input data, reflecting a particular realisation of the parameter space of interest.

The described issues are discussed further in the context of the application of physics-informed neural networks (PINNs), which have become especially popular after the publication of work [1]. The ability of such networks to solve problems with parameters is investigated in many studies and remains an urgent issue [1,2,3,4,5,6,7,8,9]. In particular, the construction of parametric models is an integral part of surrogate modelling [4,5,7,10]. In [5], the importance of constructing parametric solutions in comparison with classical numerical ones is emphasised in the sense of convenience of analysing the surrogate model under different conditions and instantaneous response when requested for any location in the spatial-parameter space of interest. The authors demonstrate how effectively solutions representing the whole families of parametric models are constructed by exploiting PINNs. It allows for solving the problem of parameter identification with high accuracy. Refining a family of parametric neural network models by minimisation the loss encoded measurement data and selecting the most suitable one is also investigated in [11,12,13].

Other relevant areas of research in the field of modelling are the multi-fidelity approaches. PINNs and multi-fidelity methods are discussed in some detail in [14]. For matching low-fidelity physics and high-fidelity sensor data, it is used transfer learning which involves updating the initial model by re-training. The multi-level method also is proposed in [6,15]. There are studies utilising active training to enhance the approximation accuracy of PINNs, for example, [6] (based on sensor data) and [5] (based on finite element simulations).

In this article, a new class of multi-fidelity physics-informed neural networks with physics-based architecture (PBA-PINNs) is proposed. The main feature of such a type of network is that not only the training of weights but also the architecture of a network itself is based on physics. It is a multi-fidelity method where, at the first stage, the PBA model is constructed based on the analytical modification of classical numerical methods with embedded neural network modules. Note that utilising numerical methods to improve PINN models not only as additional data encoded in the loss is gaining popularity [6,16,17,18]. In the approach proposed, the issue of forming a neural network initialisation is solved while the network architecture is often manually provided [14]. Considered in this manuscript, PINNs have a simple architecture. The advantages of the structure that is easy to be understood are discussed in [9] where neural networks and interpolation polynomials are combined. In [4], this question is also considered.

With a small number of iterations of the numerical method, the proposed initial PBA model is compact but low-fidelity. At the same time, this allows quick (compare with [5]) training of a network at the next medium-fidelity step by minimising the physics-informed loss across the whole parameter space. The high-fidelity training at the third stage realises the Industry 4.0 concept of active manufacturing control encoding sensor data [19].

The performance of proposed methods is demonstrated in solving a benchmark problem, namely the stationary problem of the thermal explosion of a non-isothermal chemical reactor in the plane-parallel case [20]. The Runge–Kutta and shooting methods are widely used in modelling chemical processes [21,22,23], and it is natural to leverage the analytical modification of these methods to solve the task. In similar problems, activation energy, temperature and thermal conductivity act as measured values. The measurement of thermal conductivity in a chemical reactor is considered in the works [24,25]. Papers [26,27,28] investigate temperature measurements. Parameters considered in this study also include the measurements of the activation energy of a chemical reactor which is investigated in [29,30].

This article is structured as follows. Section 2 discusses methods applied at each stage of the approach proposed in detail. Section 3 specifies the bench problem, presents the results of constructing multi-fidelity parametric PBA-PINN models and demonstrates an application of high-fidelity networks to a parameter identification problem. Section 4 provides conclusions and a discussion of results and the method itself.

## 2. Materials and Methods

**Figure 1:**
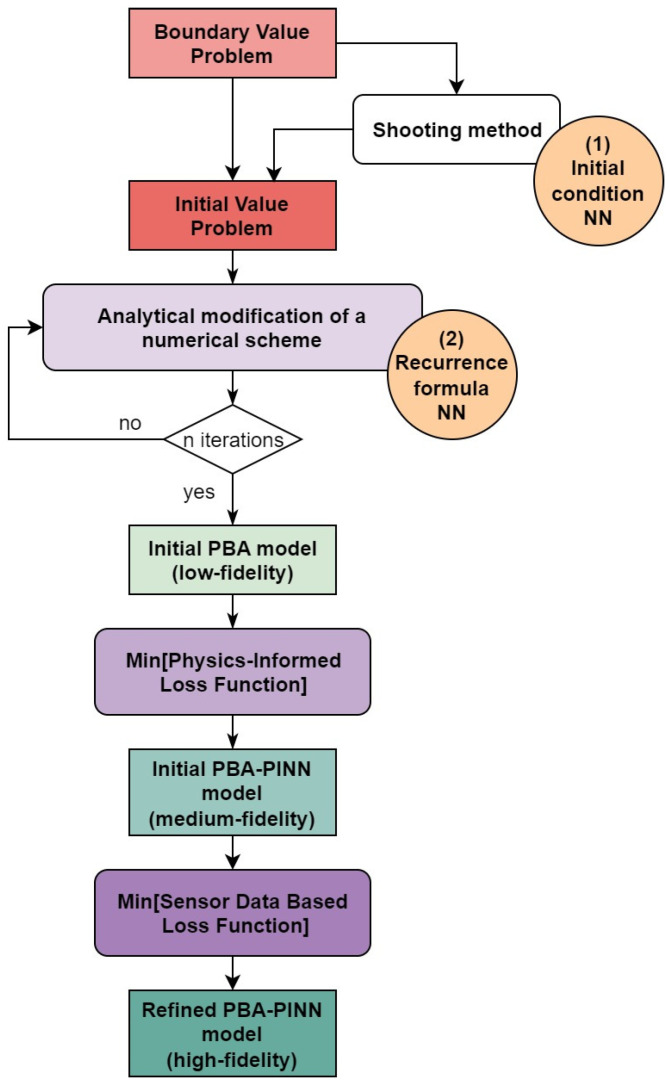
General scheme of constructing and training multi-fidelity physics-informed neural networks with physics-based architecture (PBA-PINN).

In this section, the stages of building a multi-fidelity physics-informed neural network with physics-based architecture for some boundary value problems are described sequentially. The whole process is schematically shown in Figure 1.

### 2.1. Problem Statement

Consider boundary value problems of the general form

(1) $$y^{\prime }\left(x\right)=f\left(x,y\left(x\right)\right),$$

$x\in [a_{1},a_{2}]=D$, with boundary conditions

(2) $$B_{1}\left[y\right]\left(a_{1}\right)=b_{1},\;B_{2}\left[y\right]\left(a_{2}\right)=b_{2};$$

where f(x,y(x)) is an reasonable function, $B_{1}\left[\cdot \right],B_{2}\left[\cdot \right]$ are appropriate operators and y(x) is a hidden solution.

### 2.2. Analytical Modification of the Shooting Method and Constructing PBA Model

Here, the analytical modification of the shooting method is presented. Task (1) and (2) is regarded as an initial value problem by guessing

(3) $$y\left(x_{0}\right)=y_{0},$$

where $x_{0}$ is some point on the interval *D*. Further, according to an approach described, for example, in [16], an analytical solution on the interval with a variable right (left) end x∈D is built by means of known formulas for the numerical solution of the Cauchy problem for a system of ordinary differential equations [31].

Classical numerical methods consist in dividing the interval across which the problem is solved into *n* parts $x_{0}<\ldots <x_{n}=x_{0}+a$. To find the values of an approximate solution at these points, an iterative formula

(4) $$y_{k+1}=A\left[f,y_{k},y_{k+1},h_{k},x_{k}\right],$$

where $h_{k}=x_{k+1}-x_{k}$, is used. Here, $y_{k}$ approximates the exact value of the desired solution at the point $x_{k}$ and A[·] is a function that defines the specific method leveraged. The steps $h_{k}$ are regarded as functions of a variable *x* [16], $h_{k}\left(x\right)$. In the simplest case of uniform partition, it follows that $h_{k}=\left(x-x_{0}\right)/n$ and $x_{k}=x_{0}+\left(x-x_{0}\right)k/n$.

The function $y_{n}\left(x,y_{0}\right)$ constructed at the final step determines an approximate analytical solution to the problem (1) + (3). Note, that this solution includes as a vector parameter $y_{0}=y_{0}\left(x\right)$ which, similar to the classical shooting method, is determined from the boundary conditions

(5) $$B_{1}\left[y_{n}\right]\left(a_{1},y_{0}\right)=b_{1},\;B_{2}\left[y_{n}\right]\left(a_{2},y_{0}\right)=b_{2}.$$

Thus, an analytical modification of any classical numerical scheme and the shooting method is obtained. The solution constructed in this way is a model with PBA, which can be considered as a deep neural network with *n* hidden layers. Models of this type are discussed in more detail in [16,18]. Choosing a large enough *n* provides an arbitrarily well approximation, but in this work, PBA solutions with one layer are studied due to regarding them as compact low-fidelity models.

Note that Figure 1 contains round symbols near the shooting method and analytical modification of numerical scheme blocks, indicating that there are abilities to embed neural networks at these steps.

If Formula (4) defines some explicit numerical method, then the approximate solution can be calculated as an explicit function. If it is inconvenient to use it directly, for example, a cumbersome expression, you can approximate this function using a neural network. As a result of applying the iterative Formula (4) including replacing with the neural network function an approximate solution to the problem (1) and (2), which is a multilayer neural network function, is obtained.

If the function A depends on $y_{k+1}$, the relation (4) can be considered an equation with respect to $y_{k+1}$. In the case of having an exact solution instead of Equation (4), a relation of the form

(6) $$y_{k+1}=B\left[f,y_{k},h_{k},x_{k}\right].$$

is obtained.

It allows calculating the approximate solution to the problem (1) and (2) iteratively.

If Equation (4) cannot be solved exactly with respect to $y_{k+1}$, a specially trained neural network can be used to obtain an approximate formula of the form (6). As a result, the approximate solution in the form of a deep neural network with physics-based architecture is constructed as before.

### 2.3. PBA-PINN Model Constructing

At this stage, the network with physics-based architecture

(7) $$y_{n}\left(x,y_{0},a\right),$$

where a is a vector including all the weights of neural networks embedded at the previous step, and is further trained to solve the original differential equation by minimising the loss function, as is usually the case in works devoted to physics-informed neural networks.

If the formulation of the problem (1) and (2) contains some parameters p, they are automatically included in the expression for PBA network $y_{n}\to y_{n}\left(x,y_{0},a,p\right)$ which leads to the construction of a parametric neural network solution [2].

Further training of the neural network can be carried out by minimising the loss function

(8) $$\sum_{j=1}^{m}\left|\right|y_{n}^{\prime }\left(x_{j},p_{j}\right)-f\left(x_{j},y_{n}\left(x_{j},p_{j}\right),p_{j}\right){\left|\right|}^{2}+\lambda \left(\left|\right|B_{1}\left[y_{n}\right]\left(a_{1}\left(p_{j}\right),y_{0}\left(p_{j}\right)\right)-b_{1}\left(p_{j}\right){\left|\right|}^{2}+\left|\right|B_{2}\left[y_{n}\right]\left(a_{2}\left(p_{j}\right),y_{0}\left(p_{j}\right),p_{j}\right)-b_{2}\left(p_{j}\right){\left|\right|}^{2}\right).$$

In this case, the parameter values are taken from the area of interest. λ is a usual hyperparameter regularising the contribution of each loss term to the value of the loss function. For brevity, weights a are omitted in the entry.

The result is an approximate solution in the form of a deep physics-informed neural network with physics-based architecture. It is important to note that according to the methods proposed, the learning process starts not with a random neural network weight initialisation, but with a relatively successful initial approximation (the accuracy of approximation depends on the accuracy of the numerical method and the number of iterations used) which greatly reduce the training time of the neural network as it is demonstrated in the case study.

In the process of training a neural network, dependence $y_{n}\left(x,y_{0},p\right)$ on $y_{0}$ may be broken. It can be avoided if $y_{0}$ in Equation (5) is substituted with another neural network, the weights of which are determined in the process of minimising the appropriate error function.

### 2.4. High-Fidelity Refinement PBA-PINNs Based on Sensor Data

In the third stage, the PBA-PINN model built is further trained according to high-fidelity data coming from sensors. The compactness of the constructed model makes it convenient to adapt it to real measurements. The PBA-PINN weights are re-trained by minimising the loss function in the form

(9) $$\sum_{j=1}^{M}{\left(y_{n}\left(x_{j},y_{0},a,p_{j}\right)-m_{j}\right)}^{2},$$

where ${\left\{m_{j},p_{j}\right\}}_{j=1}^{M}$ are sensor data in points $x_{j}$.

The resulting PBA-PINN is regarded as the multi-fidelity model of an object described by differential Equations (1) and (2) and sensor data.

### 2.5. Data-Driven Parameter Identification

As it is noted before, constructing parametric solutions is important in the sense of convenience of analysing the surrogate model under different conditions and instantaneous response when requested for any location in the spatial-parameter space of interest. Thus, this model can be applied to solve an inverse problem (parameter identification) based on a few sensor data. In this case, it is proposed to identify a new parameter value using a parametric high-fidelity PBA-PINN model constructed at the previous step and obtain the predicted value by minimising the loss function of type

(10) $$\sum_{j=1}^{L}{\left(y_{n}\left(x_{j},y_{0},a,p\right)-m_{j}\right)}^{2},$$

where ${\left\{m_{j}\right\}}_{j=1}^{L}$ are sensor data in points $x_{j}$.

In the next section, the performance of the proposed methods is demonstrated in solving a benchmark problem, namely the stationary problem of the thermal explosion of a non-isothermal chemical reactor in the plane-parallel case.

## 3. Case Study

### 3.1. Problem Statement

The methods described above have been applied to the stationary problem of the thermal explosion of a non-isothermal chemical reactor in the plane-parallel case [20] under the assumption that the reaction is one-stage, irreversible, not accompanied by phase transitions, proceeds in a stationary medium. In dimensionless form, this problem can be written as a nonlinear differential equation with boundary conditions that are given by

(11) $$\left\{\begin{array}{c} \frac{d^{2}\theta }{dx^{2}}+\delta \exp\left(\theta \right)=0,\\ \frac{d\theta }{dx}\left(0\right)=0,\;\theta \left(1\right)=0, \end{array}\;\;x\in \left[0,1\right],\delta \in \left[0.2,0.8\right].\right.$$

This problem has a ground-truth solution that can be obtained by using the standard method of reducing an order [31]. This allows evaluating the quality of a solution built utilising the methodology considered above.

The variable change interval is taken from the problem statement and is associated with the transition to a dimensionless coordinate. The parameter space is selected for computational experiments based on the following considerations. For a small value of δ, an approximate solution can be obtained based on standard methods of asymptotic expansions. In addition, to achieve a small relative error at these parameter values, changing the loss function is required. Such changes are task-specific and make it difficult to use this problem to illustrate the general methodology. There is no exact solution to the problem at δ>δ∗≈0.878458. As the solution approaches this critical boundary, it becomes unstable, and the problem acquires stiff properties. The relevant problems have been left aside not to lose clarity to illustrate the general methods. In addition, when operating a real reactor, there is a tendency to avoid working close to the stability boundary. A small value of the parameter corresponds to a low reaction rate which makes the operation of the reactor ineffective. Therefore, the parameter change interval similar to the one under consideration seems to be the most interesting from a practical point of view.

### 3.2. Transformation of Equations

Reduce (11) to a system

(12) $$\left\{\begin{array}{c} \frac{d\theta }{dx}=z,\\ \frac{dz}{dx}\left(0\right)=-\delta \exp\left(\theta \right), \end{array}\;\;x\in \left[0,1\right].\right.$$

and apply the modification of the implicit Euler method, then

(13) $$\left\{\begin{array}{c} \theta_{k+1}=\theta_{k}+h_{k}z_{k+1},\\ z_{k+1}=z_{k}-h_{k}\delta \exp\left(\theta_{k+1}\right). \end{array}\right.$$

It is obtained by applying a single-layer formula (k=1) that

(14) $$\theta_{1}=\theta_{0}+\left(x-x_{0}\right)z_{0}-{\left(x-x_{0}\right)}^{2}\delta \exp\left(\theta_{1}\right).$$

Let $x_{0}=0$ then $\frac{d\theta }{dx}\left(0\right)=0$ and θ(1)=0 imply $z_{0}=0$ and $\theta_{0}=\delta$. Therefore,

(15) $$\theta_{1}=\delta -x^{2}\delta \exp\left(\theta_{1}\right).$$

This implicit equation is solved using a neural network according to the methods of embedding neural network elements described earlier.

### 3.3. Embedding Neural Network in PBA Solution

Consider an implicit equation

(16) y+sexp(y)=t.

An approximate solution y(s,t) to (16) is looked for in the form of a neural network with one hidden layer and *n* neurons per it, which can be expressed as

(17) $$\hat{y}\left(s,t,{\left\{c_{i},a_{i}\right\}}_{i=1}^{n}\right)=c_{1}+\sum_{i=2}^{n}c_{i}v\left(s,t,a_{i}\right),$$

where

(18) $$v\left(s,t,a\right)=th\left(a_{1}s+a_{2}\right)th\left(a_{3}t+a_{4}\right)$$

is an activation function, parameters ${\left\{c_{i},a_{i}\right\}}_{i=1}^{n}$ are learned by minimising the squared error loss

(19) $$J=\sum_{j=1}^{M}{\left(\hat{y}\left(s_{j},t_{j},{\left\{c_{i},a_{i}\right\}}_{i=1}^{n}\right)+s_{j}\exp\left(\hat{y}\left(s_{j},t_{j},{\left\{c_{i},a_{i}\right\}}_{i=1}^{n}\right)\right)-t_{j}\right)}^{2}.$$

Throughout this work, in the training process, inputs are resampled after 3–5 steps of nonlinear optimisation of a corresponding loss function in the domain of interest. This resampling [32] is regarded as the regularisation aimed to avoid over-fitting. In this case, input points ${\left\{s_{j},t_{j}\right\}}_{j=1}^{M}$ are from domain 0<s<t<1.

The resulting neural network is used as an approximate solution to Equation (11), namely

(20) $$\theta_{1}\left(x\right)=\hat{y}\left(x^{2}\delta ,\delta ,{\left\{c_{i},a_{i}\right\}}_{i=1}^{n}\right).$$

Further, results obtained for various *n* are considered and discussed. Table 1 shows that along with the rise in the number of neurons, the accuracy of the implicit Equation (16) solution increases as expected. Simultaneously, the accuracy of the original problem solution decreases. It is obviously related to the large error of the Euler method on the basis on which the solution is built. In addition, a network with n=20 neurons has no significant advantages in the accuracy of the corresponding solution of the implicit equation. This is due to the fact that training all networks takes the same number of epochs (2000) to avoid the bias of comparison, and longer training is required to learn a network with 20 neurons of a hidden layer.

**Figure 2:**
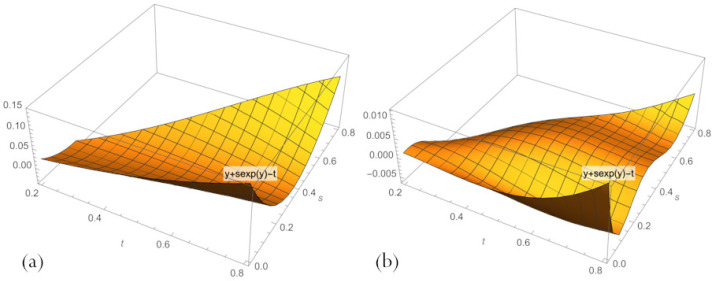
Errors in PBA solutions (17) to Equation (16), across domain 0<s<t<1, for n=3 (**a**) and n=10 (**b**).

**Figure 3:**
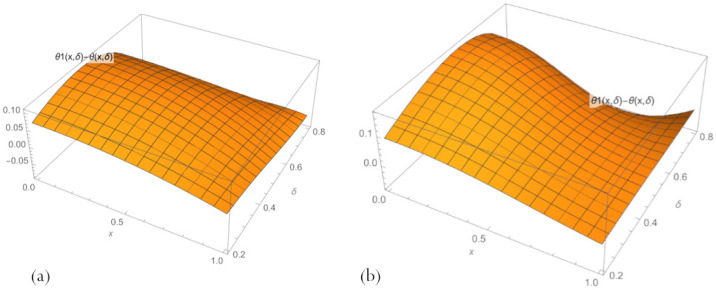
Errors in the multilayer solutions (20) with an embedded neural network (17) to problem (11), across parameter space 0.2<δ<0.8, for (**a**) n=3 and (**b**) n=10.

The errors of neural network solutions to problems (16) and (11) in the case of n=3 and n=4 are presented in the form of graphs in Figure 2 and Figure 3. Plots in Figure 2 demonstrate that a network with n=3 neurons of a hidden layer gives a much poor quality of the approximation to the exact solution to the implicit equation because the error significantly deviates from 0 in most of the input domain. A network with n=10 gives greater accuracy and has big deviations from 0 only for small *s* and big *t*. Note that in this case, the decision surface has a more tortuous character compared to the decision surface of a network with n=3 neurons of a hidden layer.

Figure 3 shows that the maximum error is reached at the left end of the parameter interval and the medium value of δ. At the same time, the error for a network with n=10 neurons is slightly higher. It is caused by inaccuracies introduced by Formula (14).

**Figure 4:**
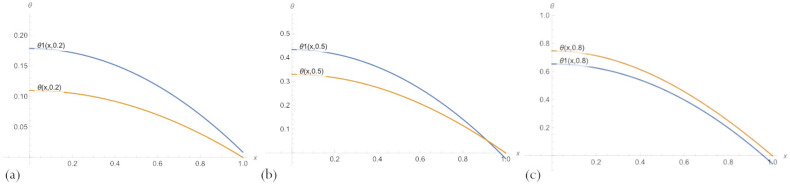
A comparison of PBA solutions (20) with an embedded neural network, $\theta_{1}\left(x,\delta \right)$, n=3 neurons, and an exact solution of the problem (11) for parameter values: (**a**) δ=0.2; (**b**) δ=0.5; (**c**) δ=0.8.

For fixed parameter δ values, several graphs of the solutions of the type (20) and the exact solution to the problem (11) are shown in Figure 4. The graphs presented in Figure 3 show that despite a significant error, the PBA solutions with an embedded neural network match the overall trend of the ground-truth solution, which allows for obtaining more accurate solutions by further training.

**Figure 5:**
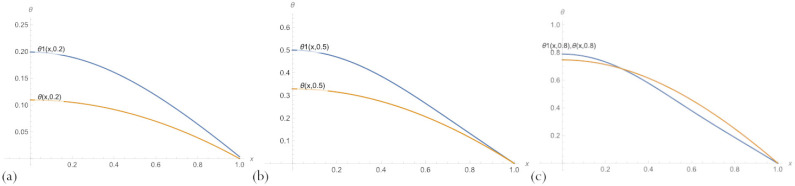
A comparison of the multilayer solutions (20) with an embedded neural network, $\theta_{1}\left(x,\delta \right)$, n=10 neurons, and an exact solution of the problem for parameter values: (**a**) δ=0.2; (**b**) δ=0.5; (**c**) δ=0.8.

Comparison of Figure 4 and Figure 5, where the same graphs for the PBA solution with n=10 are presented, shows that the solution (20) with n=3 neurons per a hidden layer of embedded network does not have such an advantage over the one with n=10, as it might seem from Table 1.

Moreover, comparing any accuracy of solutions presented in this subsection is rather conditional, since these solutions are only a qualitatively correct approximated blank for further high-fidelity classical PINN learning to effectively refine the low-fidelity initial PBA solutions with an embedded neural network.

### 3.4. Physics-Informed Refinement of Initial PBA Neural Networks

Here, the low-fidelity initial PBA solutions with an embedded neural network constructed in a previous subsection are refined. Parameters ${\left\{c_{i},a_{i}\right\}}_{i=1}^{n}$ of a parametric family of neural networks $\theta_{1}\left(x\right)=y\left(x^{2}\delta ,\delta ,{\left\{c_{i},a_{i}\right\}}_{i=1}^{3}\right)$ can be learned by minimising the loss function

(21) $$J_{1}=\sum_{j=1}^{m}\left[{\left(\theta_{1}^{\prime \prime }\left(x_{j}\right)+\delta_{j}\exp\left(\theta_{1}\left(x_{j}\right)\right)\right)}^{2}+\lambda \left({\left(\theta_{1}^{\prime }\left(0\right)\right)}^{2}+{\left(\theta_{1}\left(1\right)\right)}^{2}\right)\right].$$

The points $\left\{x_{j},\delta_{j}\right\}$ are resampled after 3–5 steps of nonlinear optimisation of the loss function (21) across domain [0,1]×[0.2,0.8].

The results of computational experiments presented in Table 2 demonstrate that neural networks proposed in this paper have a significant potential for refining by minimising the loss (21) encoding the initial formulation of the problem (11). For comparison, the results of classical neural network models with activation functions of the form (18) are presented.

### 3.5. Additional Embedding Neural Network in PBA Solution

As a further development, equality $\theta_{0}$ has been replaced with a neural network function with one hidden layer

(22) $$\theta_{0}\left(\delta ,{\left\{b_{i}\right\}}_{i=1}^{N}\right)=\sum_{i=2}^{N}c_{i}th\left(b_{i}^{1}\delta +b_{i}^{2}\right).$$

The resulting neural network has two hidden layers and is expressed as

(23) $$\theta_{1}\left(x\right)=\hat{y}\left(x^{2}\delta ,\theta_{0}\left(\delta ,{\left\{b_{i}\right\}}_{i=1}^{N}\right),{\left\{c_{i},a_{i}\right\}}_{i=1}^{n}\right).$$

where weights ${\left\{a_{i}\right\}}_{i=1}^{N}$, ${\left\{b_{i}\right\}}_{i=1}^{N}$ are trained by minimising the loss function (21).

Comparing the error values in Table 2 and Table 3, it can be concluded that a two-layer network has a significant advantage.

**Figure 6:**
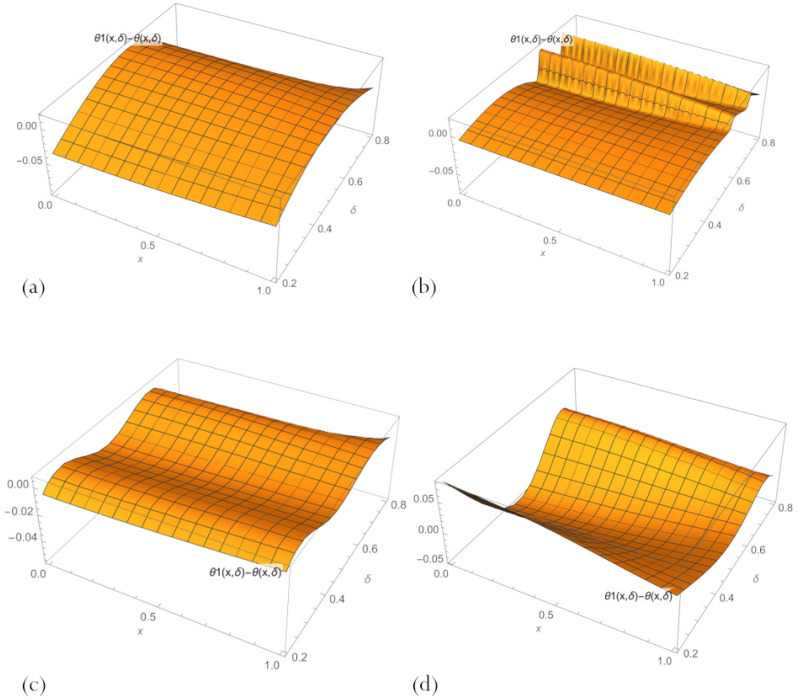
Errors in PBA-PINN (20) for (**a**) n=3 and (**b**) n=10, (**c**) in PBA-PINN (23) for n=3, N=1, and (**d**) in classical PINN with n=20 neurons of a hidden layer and activation function (18), across parameter space 0.2<δ<0.8, after learning by minimising the loss (21).

The error field in various PINN solutions to Equation (11) across the whole parameter region of interest, 0.2<δ<0.8, learned by minimising the loss (21) are shown in Figure 6. These plots make it clear that none of the PINN solutions has a big advantage over the others. Refined PBA PINN (20) with n=10 neurons has a slightly smaller error than the one with n=3 but has excessive fluctuations in the area of large δ. PBA PINN (23) with n=3, N=1 has the smallest error and matches the trend of the exact solution. A classical PINN with n=20 neurons of a hidden layer and activation function (18) has the largest error with the maximum amplitude of oscillations.

**Figure 7:**
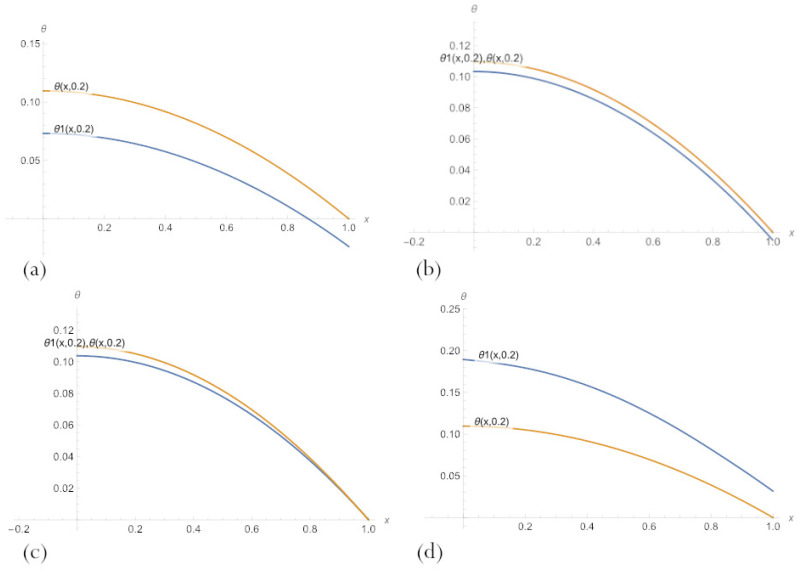
A comparison of PBA-PINN (20) for (**a**) n=3 and (**b**) n=10, (**c**) PBA-PINN (23) for n=3, N=1, and (**d**) classical PINN with n=20 neurons of a hidden layer and activation function (18), after learning by minimising the loss (21) and an exact solution to the problem (11), for δ=0.2.

**Figure 8:**
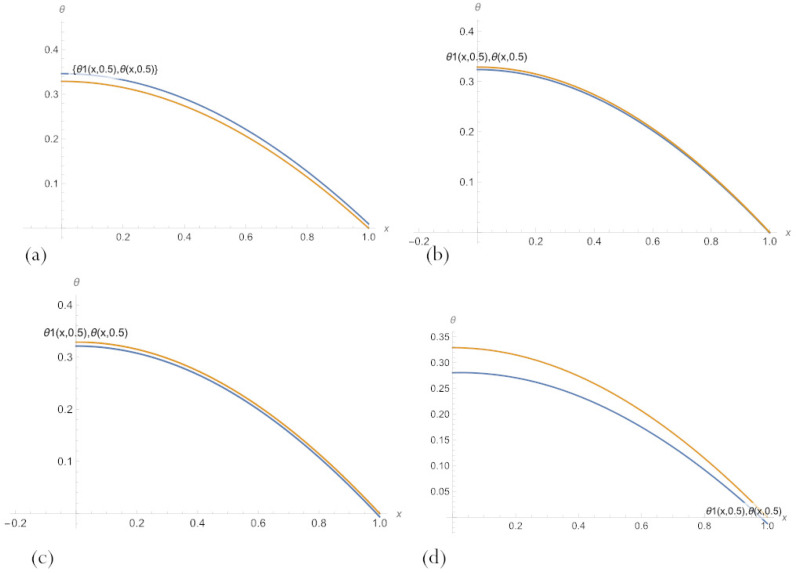
A comparison of PBA-PINN (20) for (**a**) n=3 and (**b**) n=10, (**c**) PBA-PINN (23) for n=3, N=1, and (**d**) classical PINN with n=20 neurons of a hidden layer and activation function (18), after learning by minimising the loss (21), and an exact solution to the problem (11) for δ=0.5.

**Figure 9:**
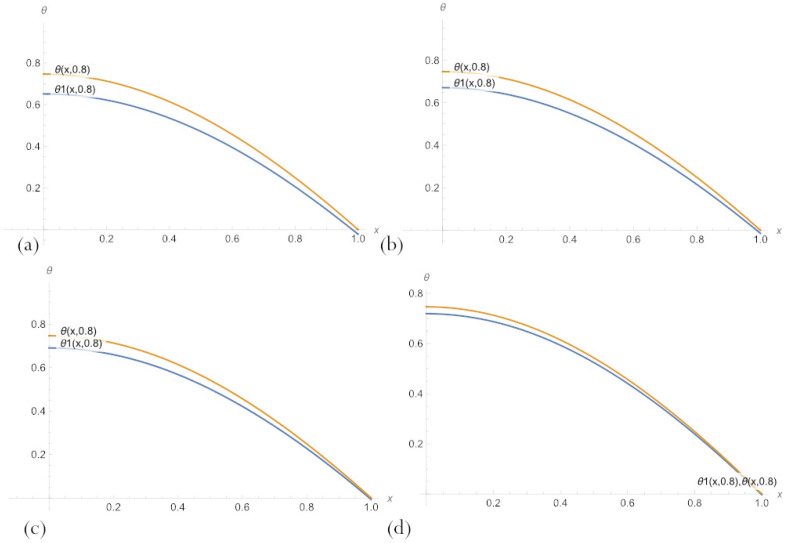
A comparison of PBA-PINN (20) for (**a**) n=3 and (**b**) n=10, (**c**) PBA-PINN (23) for n=3, N=1, and (**d**) classical PINN with n=20 neurons of a hidden layer and activation function (18), after learning by minimising the loss (21), and an exact solution to the problem (11) for δ=0.8.

Compare the results for fixed values of the parameter δ by visually presenting their graphs. Figure 7 demonstrates that, for small δ, PBA-PINN (20) with n=10 neurons and PBA-PINN (23) with n=3, N=1 have the minimum error, classical PINN with n=20 neurons of a hidden layer has the largest error. According to Figure 8, in the middle of parameter space, PBA-PINN (23) with n=3, N=1 have the minimum error, classical PINN with n=20 neurons has the largest error. At the same time, the relative errors for all networks are significantly less than errors for δ=0.2. Figure 9 shows that for big δ classical PINN with n=20, neurons have the smallest error, and the quality of other PINNs is approximately the same. It is clear from Figure 7, Figure 8 and Figure 9 that it is most convenient to have a family of PINN solutions and choose from them the most appropriate in a particular situation.

### 3.6. Data-Driven PBA-PINN Model Refinement and Discovery

The high-fidelity sensor data is leveraged to effectively refine PBA-PINN solutions to problems (11). The portability of PBA-PINN (20) with n=3 per hidden layer makes it convenient to adapt it to real measurements. Recall that this network weight has been already trained by minimising the loss function (21).

#### 3.6.1. Parametric PBA-PINN

**Figure 10:**
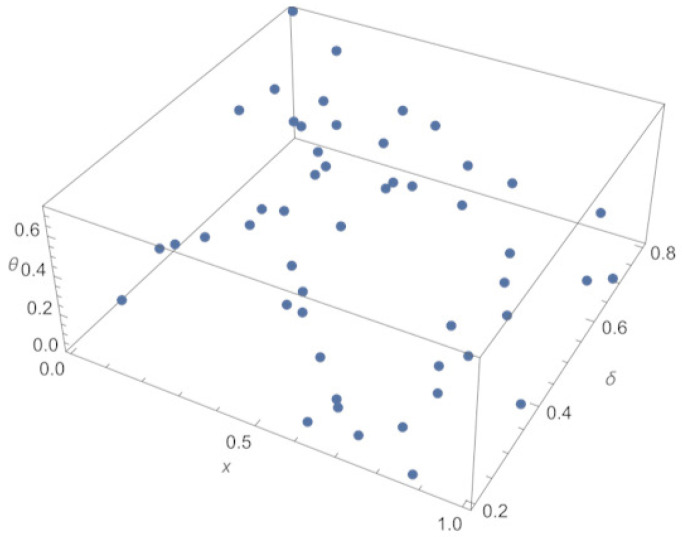
3-D plot of a training sample for refining parametric PBA-PINN, across parameter space 0.2<δ<0.8.

In the first computational experiment, M=50 random (uniformly distributed) locations ${\left\{x_{j}^{\prime },\theta_{j}^{\prime }\right\}}_{j=1}^{M}$ in spatial-parameter domain (0,1)×(0.2,0.8) have been used as input and correspondent synthetic measurements $\theta_{j}^{\prime }$ calculated by means the exact solution to (11) as output. The training sample is shown in Figure 10. It can be seen that random sampling is used without the intention of covering the whole domain of interest evenly.

For training, the loss encoding generated sensor data ${\left\{x_{j}^{\prime },\delta_{j}^{\prime },\theta_{j}^{\prime }\right\}}_{j=1}^{M}$ is utilised, namely

(24) $$J_{2}^{\prime }=\sum_{j=1}^{M}{\left(\theta_{1}\left(x_{j}^{\prime },\delta_{j}^{\prime }\right)-\theta_{j}^{\prime }\right)}^{2}.$$

**Figure 11:**
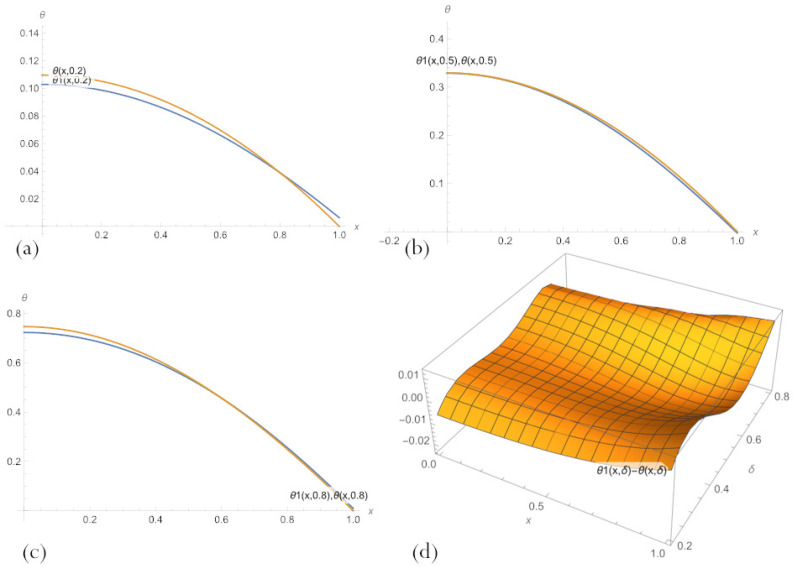
A comparison of parametric PBA-PINN (20), $\theta_{1}\left(x,\delta \right)$, n=3, after two consecutive training weights by minimising the losses (21) and (24), respectively, and an exact solution to the problem for parameter values: (**a**) δ=0.2; (**b**) δ=0.5; (**c**) δ=0.8; and (**d**) error in this PBA-PINN across parameter space 0.2<δ<0.8.

**Figure 12:**
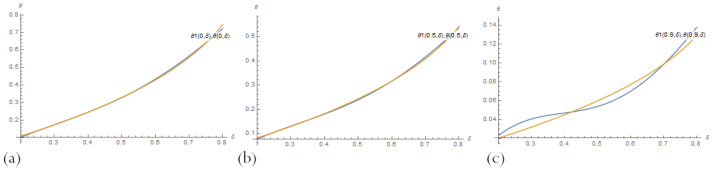
A comparison of parametric PBA-PINN (20), $\theta_{1}\left(x,\delta \right)$, n=3, after two consecutive training weights by minimising the losses (21) and (24), respectively, and an exact solution to the problem for spatial locations: (**a**) x=0; (**b**) x=0.5; (**c**) x=0.9.

Figure 11 illustrates the results of training PBA-PINN (20) by minimising the loss (24). A comparison of them with graphs in Figure 7a, Figure 8a and Figure 9a shows drastic improvement in the quality of PBA-PINN solution, especially at the ends of parameter interval. Figure 12 displays PBA-PINN solutions for fixed spatial locations.

#### 3.6.2. PBA-PINN for Fixed Parameter Value

In the next experiment, parameter δ is fixed on the (0.2,0.8) and 10 random inputs $x_{j}^{\prime \prime }$ of PBA-PINN (20) are taken on [0,1]. Correspondent synthetic measurements $\theta_{j}^{\prime \prime }$ calculated as before.

For training, the loss encoding generated sensor data ${\left\{x_{j}^{\prime \prime },\delta ,\theta_{j}^{\prime \prime }\right\}}_{j=1}^{M}$ is utilised, namely

(25) $$J_{2}^{\prime \prime }\sum_{j=1}^{M}{\left(\theta_{1}\left(x_{j}^{\prime \prime },\delta \right)-\theta_{j}^{\prime \prime }\right)}^{2}.$$

The results of computational experiments presented in Table 4 demonstrate that errors after additional training PBA-PINN according to the data for the entire spatial-parameter space are several times less than errors presented in Table 2.

#### 3.6.3. Parameter Identification

**Figure 13:**
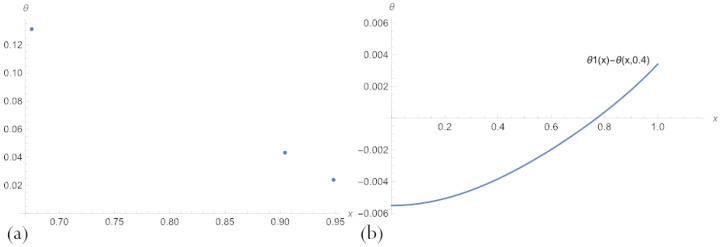
Parameter identification and model discovery. (**a**) A sample with K=3 synthetic measurements; (**b**) error in corresponded parametric PBA-PINN solution (20) to (11) for predicted parameter δ value.

**Figure 14:**
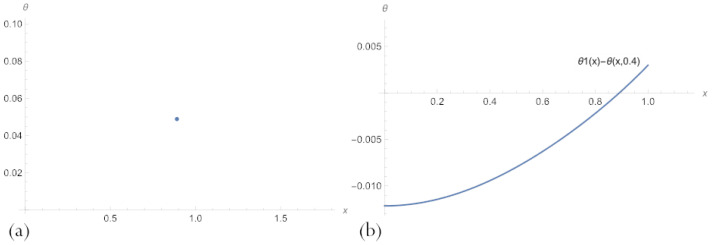
Parameter identification and model discovery. (**a**) A sample with K=1 synthetic measurement; (**b**) error in corresponded parametric PBA-PINN solution (20) to (11) for predicted parameter δ value.

Consider a slightly different problem that shows the possibilities of using parametric data-driven trained PBA-PINN. It is demonstrated by applying this network to an inverse problem, namely, predicting the δ parameter value which corresponds to a certain sensor data. As it has been done before, synthetic temperature measurements ${\left\{x_{j}^{\prime \prime \prime },\theta_{j}^{\prime \prime \prime }\right\}}_{j=1}^{K}$ at *K* random points on the interval [0,1] are calculated for δ=0.4 by means of the exact solution to the problem in question. The training samples used in the experiments and corresponding PBA-PINN solutions are shown in Figure 13a and Figure 14a.

The PBA-PINN with n=3 refined by two training is regarded as a final parametric model $\theta_{1}\left(x,\delta \right)$ satisfying to Equation (11) across spatial-parameter domain (0,1)×(0.2,0.8). The unknown δ is obtained by minimising the loss

(26) $$J_{3}^{\prime \prime }=\sum_{j=1}^{K}{\left(\theta_{1}\left(x_{j}^{\prime \prime \prime },\delta \right)-\theta_{j}^{\prime \prime \prime }\right)}^{2}.$$

The parameter values predicted as a result of querying the parametric PBA-PINN model are presented in Table 5.

It is clear from Figure 14b that utilising an inaccurate PBA-PINN solution with only n=3 neurons and one measurement from sensors allows predicting a sufficiently high-quality approximate solution in the case of unknown parameter δ. Compare with [7] where, to perform model inversion, 100 points are used.

## 4. Discussion

This manuscript proposes a new class of physics-informed neural networks, PBA-PINN. The main feature of this type of network is that not only the training of weights but also the architecture (structure) of the network itself is based on physics.

The task of training a neural network by minimising the loss function encoding measurement data, differential equations and boundary conditions is well-known and investigated. However, the issue of forming a good initial approximation to the weights of a neural network (initialisation), and especially the question of selecting a network architecture, that meets the features of the problem being solved, has not been sufficiently investigated. The paper puts forward the method for solving these issues based on the use of differential equations and, accordingly, the physics of processes occurring in a simulated object.

The process of building and utilising these kinds of networks proceeds in the following three stages. In the first step, based on the analytical modification of classical numerical methods, the task of constructing an approximate neural network solution of a boundary value problem for a differential equation is reduced to the construction of an approximate solution with a physics-based architecture. To simplify the solution of an equation (explicit or implicit) at each iteration of the numerical method, this equation is proposed to be solved using a neural network. Network weights are trained in the usual way based on minimising the loss function. An essential feature of the network is that the task parameters can be among the inputs. With a small number of iterations of the numerical method, the resulting solution with physics-based architecture is compact, but low-fidelity. At the second stage, the PBA network built is further trained to solve the differential equation by minimising the loss function, as is typical in works devoted to PINNs. In this case, the network is trained not only across the set of input variables of the original problem but also across parameter space. In the third stage, the PBA-PINN model built is further trained according to data coming from sensors. By means of the resulting high-fidelity model, it is possible to solve the problems of parameter identification, equations discovery and other tasks, for example, the control problem.

The performance of the proposed methods is demonstrated on a benchmark problem of modelling processes in a chemical reactor.

The results of computational experiments have shown that for the problem in question, the proposed method allows the construction of very small physics-informed neural network models that reflect the simulated object with acceptable accuracy. The insufficient accuracy of low-fidelity models constructed at the first stage is compensated by the possibility of refining the models through additional high-fidelity training at the second and third stages. The results of this multi-fidelity training PBA-PINNs have been compared with the results of training classical PINNs. The proposed PBA-PINNs have allowed for reaching several times greater accuracy than the standard fully-connected neural networks. Data-driven computational experiments have demonstrated that the proposed parametric low-fidelity models are suitable for subsequent retraining and solving problems of parameter identification based on measurement data. In work [33], the same parametric differential problem was solved by applying the analytical modification of such numerical methods as the corrected Euler method and the Störmer method. The authors have shown that the accuracy of a solution improves as the number of iterations increase. Note that on the basis of a similar low-fidelity model, with the help of subsequent additional training, in this article, it has been possible to obtain the parametric model with comparable accuracy. Moreover, when completing training according to data for specific parameter values, the superiority of the final high-fidelity solution has been expressed in reducing the maximum error by three orders of magnitude. This result is comparable to solutions obtained in [34,35] where similar problems with fixed parameter values were solved.

It is advisable to use the devised PBA-PINNs in the problems of surrogate modelling and modelling real objects when it is difficult or inappropriate to build a sufficiently accurate physical model and, accordingly, a mathematical model in the form of a boundary value problem for a differential equation (or a system of such equations). In this case, it is assumed that there are sensor data that can improve the accuracy of the model, but which are not enough to build a model without using differential equations.

## Figures and Tables

**Table 1:** Results of solving the implicit Equation (16). A comparison of the mean square error (MSE) and the maximum value of the absolute error for the neural network solution to Equation (16) with basis functions (18) and the corresponding solution of the problem (11) for different numbers of neurons per a hidden layer.

Number of Neurons	MSE for (16)	max|Error| for (16)	MSE for (11)	max|Error| for (11)
n=3	0.0259	0.146	0.0617	0.106
n=5	0.00354	0.0238	0.0900	0.168
n=10	0.00189	0.0117	0.0880	0.172
n=20	0.00223	0.0100	0.0875	0.176

**Table 2:** Results of PBA-PINN learning. A comparison of the mean square error (MSE) and the maximum value of the absolute error for PBA-PINN (20) with *n* neurons per a hidden layer in an embedded network and classical PINN with *n* neurons of a hidden layer and activation function (18) after learning by minimising the loss (21).

Number of Neurons	MSE for Learned (20)	max|Error| for Learned (20)	MSE for Classical PINN	max|Error| for Classical PINN
n=3	0.0192	0.0947	0.120	0.393
n=5	0.0118	0.0678	0.0448	0.149
n=10	0.0113	0.0706	0.0374	0.123
n=20			0.0269	0.0792

**Table 3:** Results of PBA-PINN learning. The mean square error (MSE) and the maximum value of the absolute error for PBA-PINNs (23) with *N* neurons per first hidden layer and *n* neurons per second one after learning by minimising the loss (21).

Number of Neurons	MSE	max|Error|
n=3, N=1	0.00865	0.0527
n=10, N=3	0.00593	0.0437

**Table 4:** The mean square error (MSE) and the maximum value of the absolute error for PBA-PINNs (20) with n=3 neurons per a hidden layer after two consecutive training weights by minimising the losses (21) and (24), respectively.

δ	MSE for (11)	max|Error| for (11)
(0.2,0.8)	0.00445	0.0163
0.2	0.0000456	0.000155
0.5	0.000148	0.000272
0.8	0.000798	0.00178

**Table 5:** The inverse problem parametric PBA-PINN (20) solving results. Predicted parameter δ values for different sensor data numbers and ground-truth value δ=0.4.

Number of Sensor Data	Predicted δ	|Error|
K=3	0.378	0.022
K=1	0.364	0.046

## Data Availability

Data available on request from the authors.
